# Gender Differences in Smartphone Addiction and Depression: Expanding Perspectives in Mental Health

**DOI:** 10.1192/j.eurpsy.2026.12038

**Published:** 2026-07-31

**Authors:** N. Pirwani, A. Vujic, A. Szabo

**Affiliations:** 1 Doctoral School of Education, Faculty of Education and Psychology, Eötvös Loránd University (ELTE), Budapest; 2 Faculty of Health and Sport Sciences, Széchenyi István University, Győr; 3 Institute of Health Promotion and Sport Sciences, Faculty of Education and Psychology, Eötvös Loránd University (ELTE), Budapest, Hungary

## Abstract

**Introduction:**

The pervasive use of smartphones in daily life has brought growing concern regarding their association with mental health issues, particularly depression. Smartphone addiction (SA) has been increasingly recognized as a behavioral addiction, with emerging evidence suggesting gender-specific vulnerability.

**Objectives:**

This systematic review aims to explore how the relationship between SA and depression differs by gender, identifying the patterns and psychosocial contributors that may explain these disparities.

**Methods:**

A systematic search was conducted across five databases: PubMed, Web of Science, Scopus, ProQuest, and Google Scholar. Sixteen studies met the inclusion criteria, comprising fifteen cross-sectional and one longitudinal study. These studies covered a diverse range of populations from regions and countries including Europe, China, Japan, South Korea, Saudi Arabia, India, and the United States. The PRISMA framework guided the review process, and study quality was assessed using the Mixed Methods Appraisal Tool (MMAT).

**Results:**

All studies reported a statistically significant association between SA and depression. Most findings indicated that women are more vulnerable to the depressive impacts of SA, often linked to their higher engagement in hedonic smartphone activities such as social networking and communication. A smaller subset of studies reported stronger effects in men, primarily associated with gaming-related smartphone use. This suggests that the nature and purpose of smartphone use may mediate gendered mental health outcomes. The overall effect size (*d* ≈ 0.37; 95% CI: 0.32–0.42) reflects a small-to-moderate effect, indicating that gender differences in the association between smartphone addiction and depression are statistically significant and of practical relevance.

**Conclusions:**

The findings highlight important gender-based differences in the link between smartphone addiction and depression. These results call for more longitudinal and experimental studies to uncover causal mechanisms and psychosocial moderators. Importantly, the review underscores the need for gender-sensitive mental health strategies and digital well-being interventions. By recognizing how smartphone addiction affects men and women differently, mental health care can be more effectively tailored, expanding the horizons of personalized and equitable care in the digital age.

**Disclosure of Interest:**

None Declared

